# Significance of apparent diffusion coefficient in diagnosis of rectal carcinoma

**DOI:** 10.3389/fonc.2024.1464183

**Published:** 2024-10-02

**Authors:** Milica Šarošković, Miloš Vuković, Stefan Stojanoski, Milica Zorić, Nataša Prvulović Bunović, Milena Spirovski, Igor Nosek

**Affiliations:** ^1^ Department of Radiology, Faculty of Medicine Novi Sad, University of Novi Sad, Novi Sad, Serbia; ^2^ Department for Radiology diagnostics, Oncology Institute of Vojvodina, Sremska Kamenica, Serbia; ^3^ Department of Nuclear Medicine, Faculty of Medicine Novi Sad, University of Novi Sad, Novi Sad, Serbia; ^4^ Department of Oncology, Faculty of Medicine Novi Sad, University of Novi Sad, Novi Sad, Serbia

**Keywords:** DWI, ADC, rectal cancer, post-radiation proctitis, TNM, lymph nodes

## Abstract

**Introduction:**

The apparent diffusion coefficient (ADC) is a quantitative parameter that facilitates the detection and reliable differentiation of rectal cancer. MR differentiation between rectal carcinoma, post-radiation proctitis, and normal rectal wall with the ADC values and their comparison depending on the level of tumor markers and pathohistological characteristics of rectal carcinoma.

**Methods:**

The retrospective study performed at the Oncology Institute of Vojvodina included 300 patients, 100 each with rectal cancer, post-radiation proctitis, and normal rectum. Mean ADC values were obtained by measuring the region of interest (ROI) of the rectal wall.

**Results:**

Rectal cancer showed lower ADC values (0.665 ± 0.086 x 10^-3^mm^2^/s) compared to both post-radiation proctitis (1.648 ± 0.268 x 10^-3^mm^2^/s) and normal rectum (1.180 ± 0.110 x 10^-3^mm^2^/s) (p<0.001). No significant differences in ADC values were observed between different grades of rectal cancer (p=0.874; p>0.05), depending on the presence of metastases in the lymph nodes (p=0.357; p>0.05), different TN stage (p=0.196; p>0.05), local spread of the tumor (p=0.312; p>0.05), the presence of RAS mutation (p=0.829; p>0.05) and the value of tumor markers (p=0.923; p>0.05). ADC values below 1.013 x 10^-3^mm^2^/s with 100% sensitivity and 96% specificity indicate the presence of rectal cancer in relation to normal wall, with a positive predictive value of 96.1% and a negative of 100%. ADC values below 1.255 x 10^-3^mm^2^/s with 100% sensitivity and 95% specificity indicate rectal cancer in relation to post-radiation proctitis. ADC values above 1.339 x 10^-3^mm^2^/s with 87% sensitivity and 89% specificity indicate post-radiation proctitis in relation to normal wall.

**Discussion:**

The ADC is a useful marker in differentiating between rectal cancer, post-radiation proctitis, and normal rectal wall with high sensitivity and specificity, but it cannot be used to distinguish the histological grades of rectal cancer, nor other pathohistological parameters.

## 1 Introduction

Rectal cancer is the third most common malignancy and is currently one of the leading cause of cancer death in humans worldwide (1, 2). Despite advances in surgical techniques, chemotherapy regimens, and radiotherapy, which have led to reductions in recurrence and mortality rates, available treatment options still vary depending on tumor stage (2).

The prognosis of rectal cancer depends on several factors, among which are the pathohistological features of the tumor, the degree of differentiation, TNM classification, the level of tumor markers, the presence of molecular pathology and many others (3).

Diffusion-weighted imaging (DWI) with the apparent diffusion coefficient (ADC) gives us more precise data as a non-invasive functional MR technique sensitive to the movement of water molecules in tissues. It has high specificity in determining tissue cellularity, distinguishing recurrence after treatment or residual tumor tissue from fibrosis or necrosis (4).

With this research, we want to emphasize the importance of the apparent diffusion coefficient both in the diagnosis of rectal cancer and in differentiating tumoral thickening of the rectal wall from post-radiation proctitis, as well as its value in differentiating such findings from normal rectal wall. Also, the aim was to determine the difference in ADC values depending on the level of tumor markers and pathohistological characteristics of rectal carcinoma, with emphasis on tumor grade, local tumor status, infiltration of lymph nodes and the presence of RAS mutation. In order to find a valuable tool for differentiation between the conditions mentioned above, we calculated cut-off values.

## 2 Material and methods

### 2.1 Subject selection

This research was a retrospective study with a total of 300 patients, whose MR images are available in the database in the period from 2013 to 2023. Patients were divided into three groups:

1. The first group consisted of 100 patients with a pathohistologically confirmed diagnosis of rectal adenocarcinoma;

2. The second group consisted of 100 patients whose MR images showed thickening of the rectal wall from post-radiation proctitis, primarily as a result of irradiation of malignancy in other anatomical locations not including the rectum;

3. The third group consisted of 100 control subjects with normal findings of the rectum on MR images.

Inclusion criteria for patients in the first group were a pathohistologically confirmed diagnosis of rectal adenocarcinoma and the existence of the first diagnostic MRI scan of the pelvis done at the Oncology Institute of Vojvodina before any therapy (chemotherapy, irradiation or a combination of the above). Inclusion criteria for patients in the second group was the thickening of the rectal wall confirmed by MR imaging as a result of irradiation of other malignancies, excluding rectal malignancy (primarily of the uterus and prostate), while the only criterion for the control group was a normal finding of the rectum described on MR imaging. The inclusion criterion for all three groups was the existence of a DWI sequence with a corresponding ADC map as a standard part of the pelvic MR protocol.

The exclusion criteria for the first group were pelvic MRI scans not performed at the Oncology Institute of Vojvodina and the use of any type of therapy for rectal cancer before the MRI scan was performed. The exclusion criterion for the second group is inflammation of the rectal wall as a result of irradiation of primary rectal cancer, as well as inflammation of the wall as part of inflammatory bowel diseases (ulcerative colitis and Crohn’s disease).

The study was approved by the institutional ethical review board and the informed consent was waived due to the retrospective manner of the study.

### 2.2 Patient data

As part of the research, the data taken from the information system of the Oncology Institute of Vojvodina were pathohistological type of tumor, values of tumor markers at the time of diagnosis, as well as the possible presence of molecular pathology findings, i.e. findings of RAS gene mutation.

### 2.3 Imaging analysis

Magnetic resonance examinations were performed on two devices: 1.5T (Siemens Aera, Erlagen, Germany) and 3T (Siemens Trio Tim, Erlagen, Germany). All patients underwent the following sequences: T1W, T2W, TIRM coronal tomograms, T1W parasagittal and T2W sagittal tomograms, T1W/T2W transverse tomograms, along with a DWI sequence with an ADC map in the transverse plane. The ADC values for all three groups were measured on the PACS system (5). The DWI sequence was analyzed to define the tumor, which was displayed as a high signal intensity corresponding to the location of the tumor mass. The ROI was manually placed on the corresponding ADC map while comparing other morphological MR sequences to ensure that the ROI was placed at the location of the primary tumor. All measurements were performed by two independent readers in consensus.

### 2.4 Statistical analysis

SPSS software version 27.0 (SPSS Inc, IBM, Armonk, NY) was used for statistical data processing. The confidence interval is 95% with a significance level of p<0.05.

The differences in the ADC values between the three groups were compared by the Kruskal-Wallis test, and between individual groups by the Mann-Whitney U test (p=0.05), because all data are continuous with an abnormal distribution that was tested by the Kolmogorov-Smirnov test. The comparison between different degrees of tumor differentiation (grades) was performed with the ANOVA test, while the difference between the individual tumor grades was performed with the t-test. The Mann-Withney U test was used for analyzing differences in the values of the ADC depending on the presence of lymph node infiltration, while the t-test was used to examine the difference in the mean ADC values between groups with elevated and normal values of tumor markers, between groups depending on the presence of RAS gene mutation, as well as patients with locally confined (T1 and T2 stage) or locally advanced tumor (T3 and T4 stage). The ADC values were also analyzed in relation to different TN stage using the ANOVA test (M stage was generally not available). An analysis of the cut-off values for ADC between the examined groups (via the ROC curve) was performed, with the determination of its sensitivity, specificity, positive and negative predictive values (p<0.001).

## 3 Results

### 3.1 Demographic data

The research included 300 subjects, 100 patients with rectal cancer, 100 patients with post-radiation proctitis and 100 subjects with normal rectal wall. In the group of patients with rectal cancer, 73 men and 27 women were examined, among whom the mean age was 64.54 ± 10.74. The second group of patients with post-radiation proctitis included 27 men and 73 women, where the mean age was 62.52 ± 9.64. There were 26 men and 74 women in the group of subjects with normal wall, with the mean age of 59.47 ± 12.52 (**Table 1**).

**Table 1:** Difference of mean ADC values between the study groups.

Groups	N	Age (mean ± SD)	ADC (mean ± SD) x 10^-3^ mm^2^/s	p-value
				<0.001
Carcinoma	100	64.54 ± 10.74	0.665 ± 0.086	
Post-radiation proctitis	100	62.52 ± 9.64	1.648 ± 0.268	
Normal wall	100	59.47 ± 12.52	1.180 ± 0.110	

### 3.2 Mean ADC values in relation to the study groups

**Figure 1:**
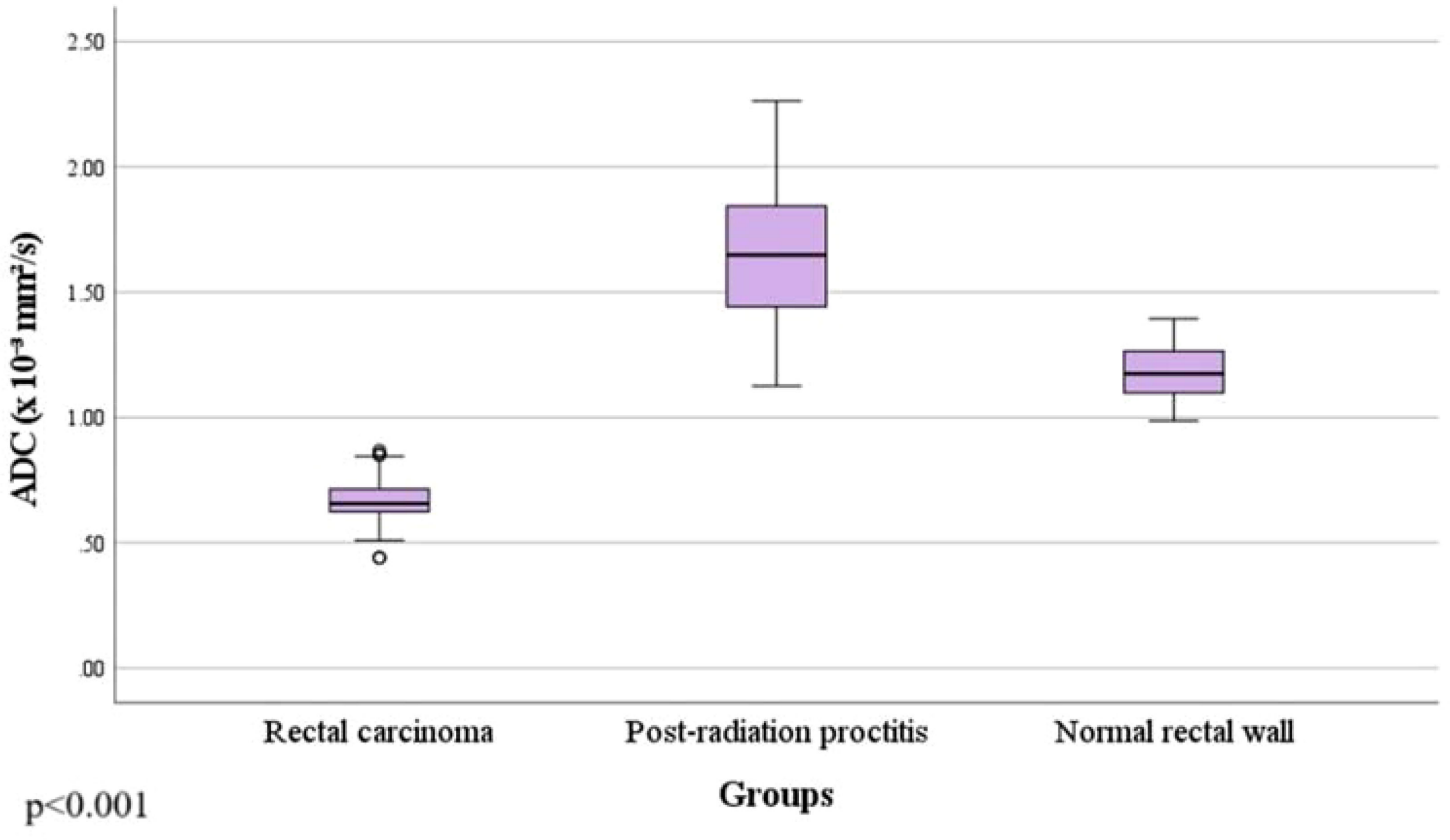
Comparison of mean ADC values between the study groups.

**Figure 2:**
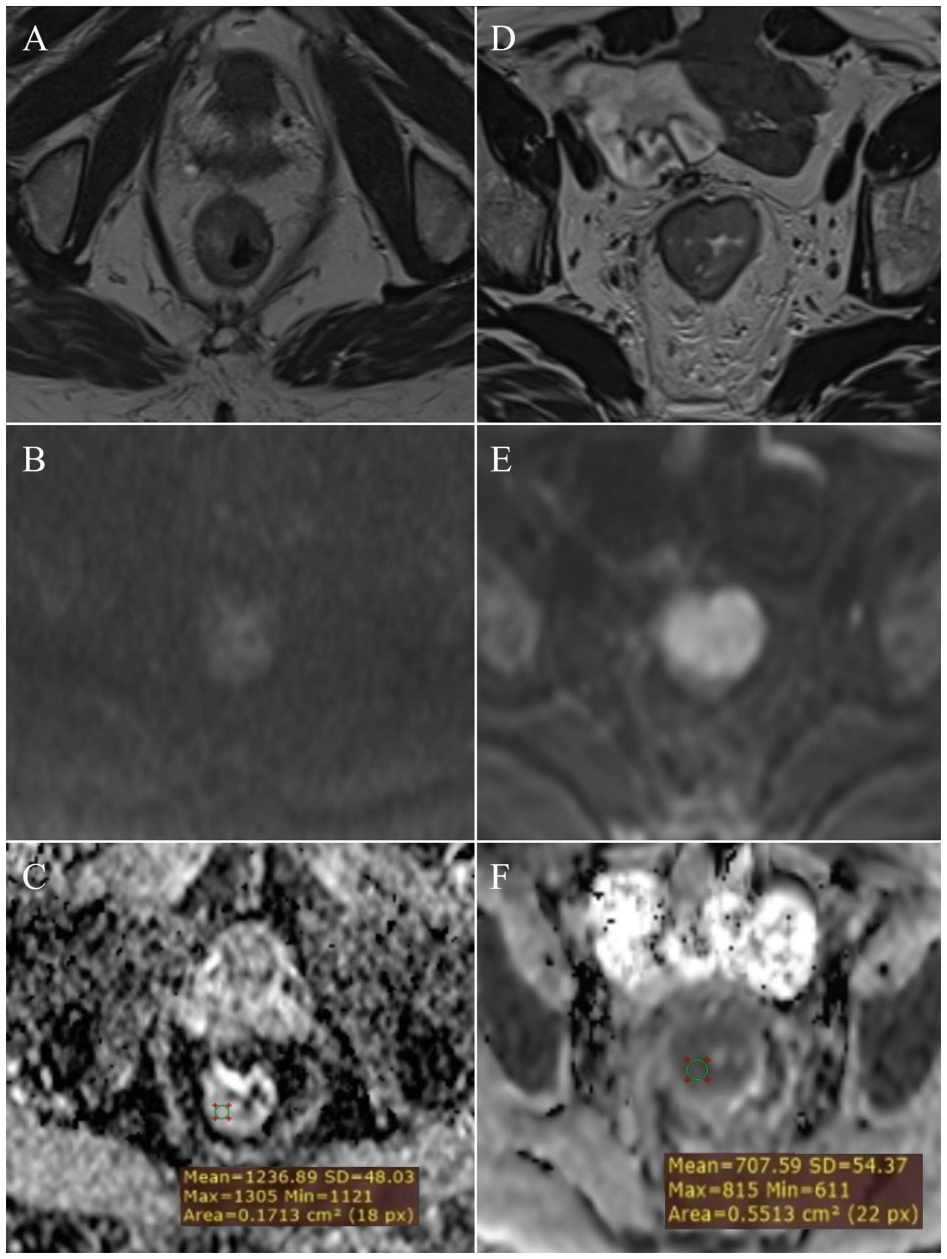
Measurement of ADC values: normal rectal wall – **(A)** (T2W), **(B)** (DWI), **(C)** (ADC map with ROI); T2 stage of rectal cancer - **(D)** (T2W), **(E)** (DWI), **(F)** (ADC map with ROI).

**Figure 3:**
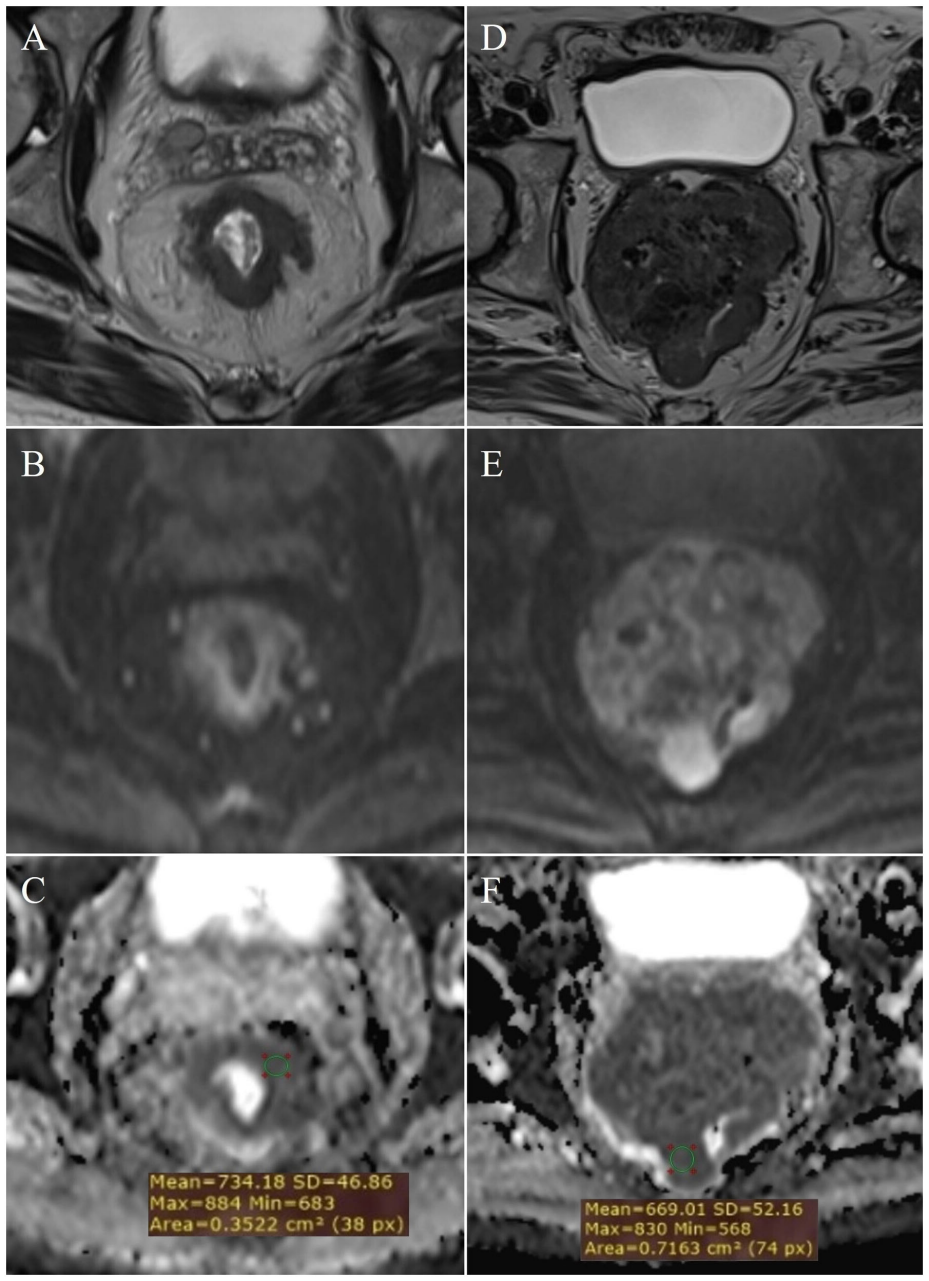
Measurement of ADC values: T3 stage of rectal cancer - **(A)** (T2W), **(B)** (DWI), **(C)** (ADC map with ROI); T4 stage - **(D)** (T2W), **(E)** (DWI), **(F)** (ADC map with ROI).

**Figure 4:**
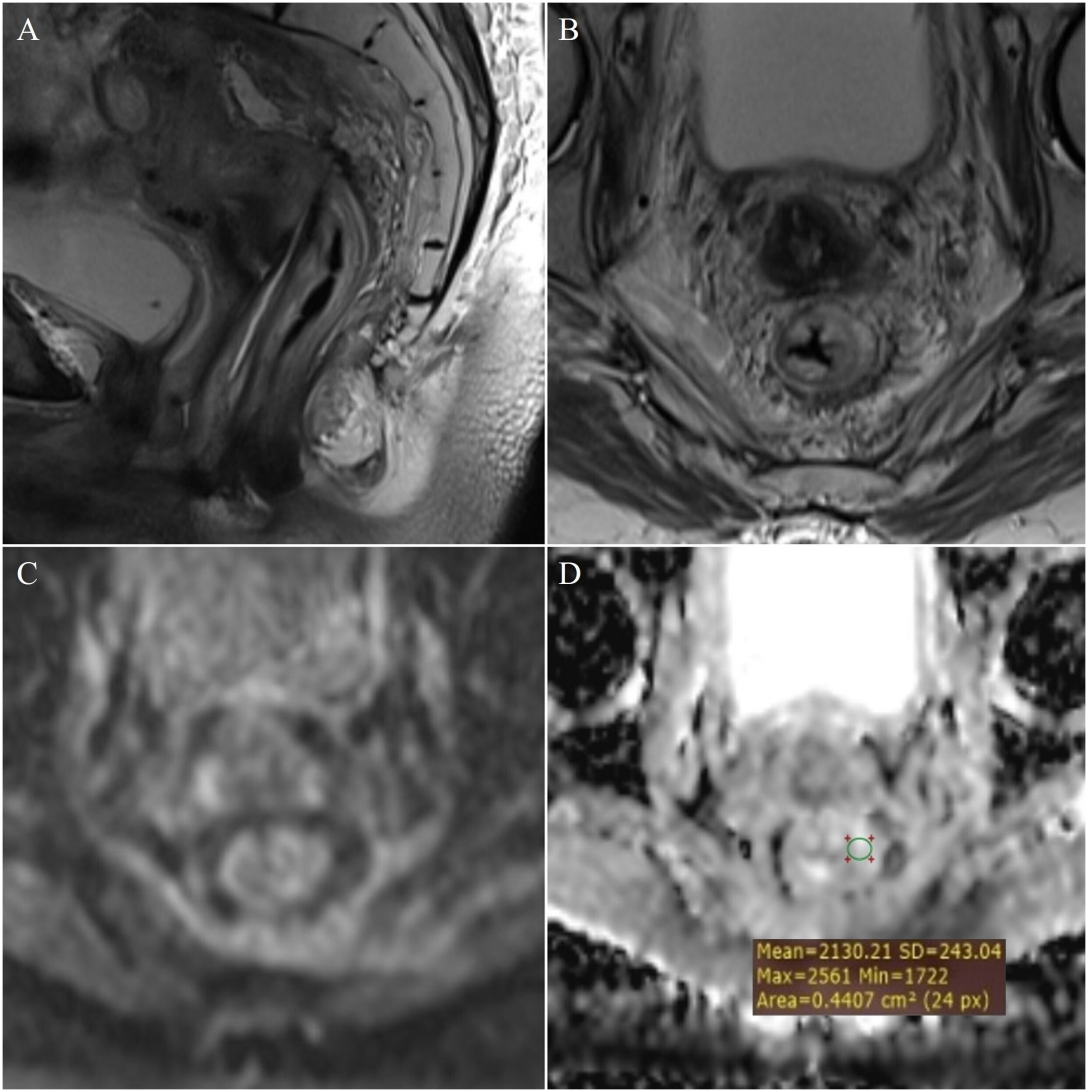
Measurement of ADC values: post-radiation proctitis - **(A)** (T2W sagittal plane), **(B)** (T2W axial plane), **(C)** (DWI) and **(D)** (ADC map with ROI).

By examining ADC values between the mentioned groups, it was determined that they differ significantly both in the whole sample and between individual groups (p<0.001) (**Figure 1**, **Table 1**). It was found that ADC values in patients with rectal cancer (**Figures 2D–F**, **3**) were statistically significantly lower both in comparison to the group of patients with normal findings (**Figures 2A–C**) and in comparison to the group of patients with post-radiation proctitis (**Figure 4**). On the other hand, in the group of patients with post-radiation proctitis, it was determined that the ADC values were significantly higher compared to the group of patients with normal findings.

### 3.3 Determining the cut-off mean ADC values to differentiate between study groups

**Table 2**. presents the estimated cut-off values for each examined group, including their corresponding sensitivity (Sn), specificity (Sp), positive predictive value (PPV), and negative predictive value (NPV) (p<0.001). The primary objective was to enhance both the sensitivity and specificity of the diagnostic test, thereby maximizing its practical utility in routine radiological practice. To accomplish this, multiple cut-off values with varying sensitivity and specificity profiles were utilized, aiming to improve the differentiation between the study groups.

**Table 2:** Cut-off mean ADC values to differentiate between study groups.

Groups	Cut-off ADC x 10^-3^ mm^2^/s	Sn^*^ (%)	Sp^*^ (%)	PPV* (%)	NPV* (%)	p-value
						<0.001
Carcinoma/normal wall (**Figure 6A**)	0.927	100	100			
	1.013	100	96	96.1	100	
Carcinoma/post-radiation proctitis (**Figure 6A**)	0.996	100	100			
	1.255	100	95	95.2	100	
Post-radiation proctitis/normal wall (**Figure 6B**)	1.339	87	89	87.9	87.2	

^* Sn, sensitivity; Sp, specificity; PPV, positive predictive value; NPV, negative predictive value.^

### 3.4 Clinical and histopathological characteristics of patients with rectal cancer

The characteristics of the patients with rectal cancer are listed in **Table 3**. Patients with a pathohistological diagnosis of adenocarcinoma were included in the study. Among them there were no T1 category patients, within the T2 category (**Figures 2D–F**) there were 26 patients, T3 (**Figures 3A–C**) 54 and T4 (**Figures 3D–F**) 20 patients. Lymph nodes were not infiltrated in 37 patients (N0 category), 1-3 lymph nodes were infiltrated in 31 patients (N1 category), and 4 or more lymph nodes were infiltrated in 32 patients (N2 category).

**Table 3:** Correlation between histological, clinical parameters and ADC values of patients with rectal cancer.

Parameters	N (%)	ADC (mean ± SD) x 10^-3^ mm^2^/s	p-value
Sex			0.332
Men	73 (73)	0.660 ± 0.083	
Women	27 (27)	0.680 ± 0.094	
Histological grade	97		0.874
Well differentiated (G1)	5 (5.1)	0.674 ± 0.059	
Moderately differentiated (G2)	85 (87.6)	0.667 ± 0.087	
Poorly differentiated (G3)	7 (7.2)	0.651 ± 0.110	
T category			0.552
T1	0 (0)		
T2	26 (26)	0.650 ± 0.072	
T3	54 (54)	0.667 ± 0.089	
T4	20 (20)	0.677 ± 0.097	
N category			0.590
N0	37 (37)	0.675 ± 0.081	
N1	31 (31)	0.653 ± 0.085	
N2	32 (32)	0.664 ± 0.094	
TN stage			0.196
T2N0	19 (19)	0.648 ± 0.069	
T2N1	6 (6)	0.632 ± 0.062	
T2N2	1 (1)	0.796 ± 0	
T3N0	15 (15)	0.713 ± 0.085	
T3N1	17 (17)	0.642 ± 0.077	
T3N2	22 (22)	0.656 ± 0.091	
T4N0	3 (3)	0.658 ± 0.079	
T4N1	8 (8)	0.694 ± 0.109	
T4N2	9 (9)	0.668 ± 0.099	
Local tumor status			0.312
Locally confined (T1 and T2)	26 (26)	0.650 ± 0.072	
Locally advanced (T3 and T4)	74 (74)	0.670 ± 0.091	
Lymph nodes			0.357
Negative (N0)	37 (37)	0.675 ± 0.081	
Positive (N1 and N2)	63 (63)	0.659 ± 0.089	
RAS status	19		0.829
Without mutation	9 (47.4)	0.647 ± 0.093	
With mutation	10 (52.6)	0.637 ± 0.098	
Tumor markers			0.923
CEA	42	0.653 ± 0.088	
<4.7 ng/ml	20 (47.6)	0.651 ± 0.072	
≥4.7 ng/ml	22 (52.4)	0.647 ± 0.108	
CA 19.9	40	0.648 ± 0.083	
<26.6 U/ml	25 (62.5)	0.641 ± 0.075	
≥26.6 U/ml	15 (37.5)	0.659 ± 0.096	

### 3.5 Mean ADC values in relation to the degree of differentiation of rectal cancer

**Figure 5:**
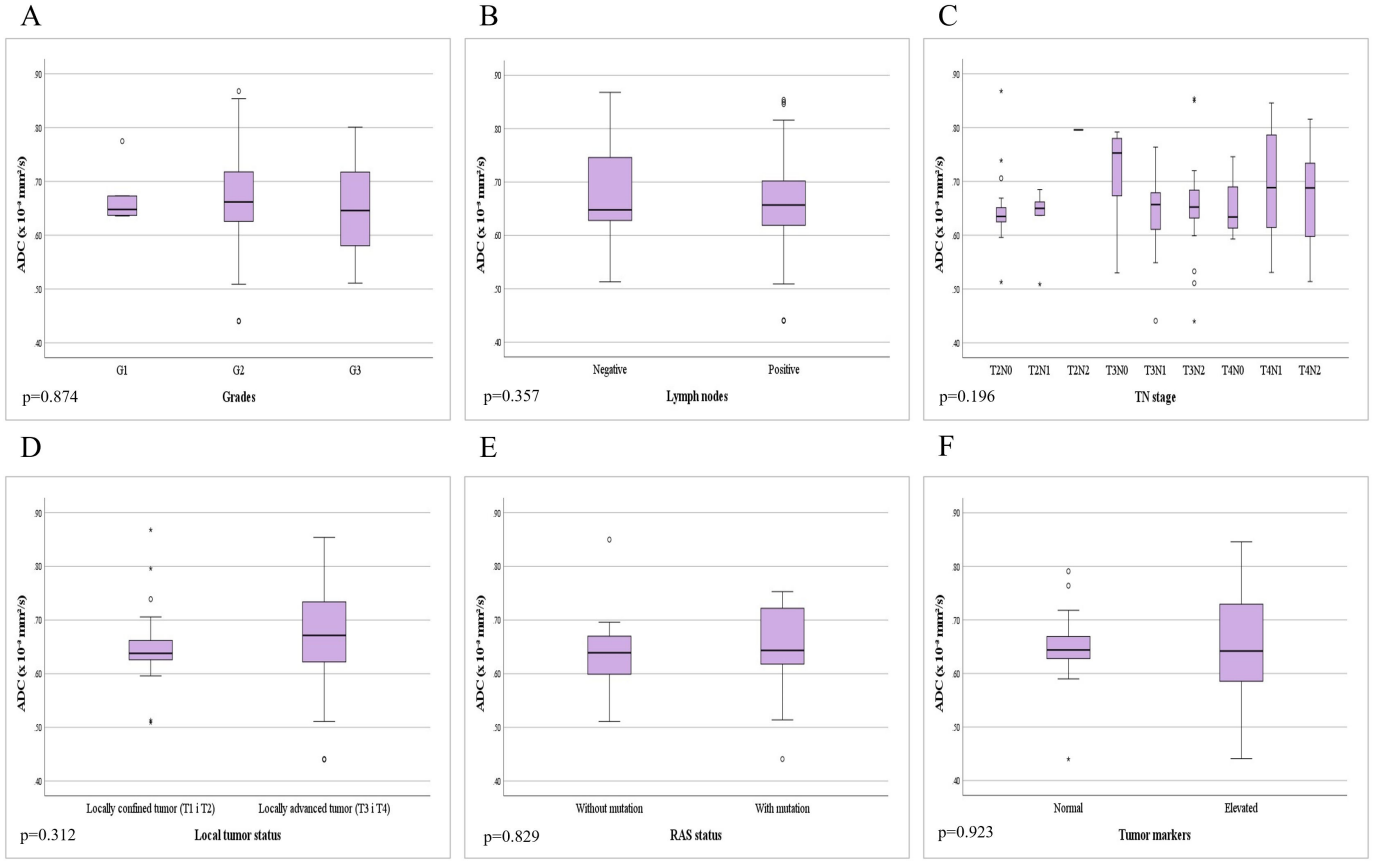
Comparison of mean ADC values depending on the: **(A)** different grades of rectal cancer; **(B)** presence of metastases in locoregional lymph nodes; **(C)** different TN stage; **(D)** local tumor status; **(E)** presence of RAS mutation; **(F)** level of tumor markers.

In patients with rectal cancer, there were 5 patients with a well differentiated tumor (G1), 85 patients with a moderately differentiated tumor (G2) and 7 patients with a poorly differentiated tumor (G3), while data on tumor grade was not available for 3 patients. Examining the ADC values between the mentioned groups we did not reveal a statistically significant difference both in the whole sample (p=0.874; p>0.05) and between individual grades (**Figure 5A**). Statistical analysis showed that ADC values between G1 and G2 tumors do not differ significantly (p=0.865; p>0.05), nor between G1 and G3 tumors (p=0.677; p>0.05), nor between G2 and G3 tumors (p=0.636; p>0.05).

**Figure 6:**
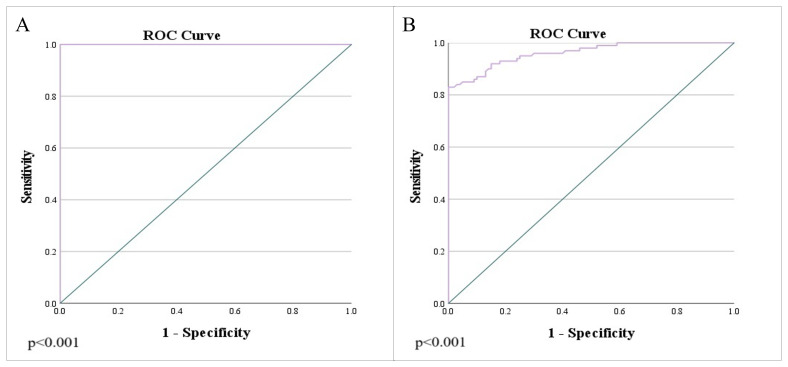
Analysis of the ROC curve of ADC values: **(A)** differentiation between rectal cancer and normal wall, as well as the differentiation of rectal cancer and post-radiation proctitis; **(B)** differentiation of post-radiation proctitis and normal wall.

### 3.6 Mean ADC values in relation to the presence of metastases in regional lymph nodes

In patients with rectal cancer, the absence of metastatic infiltration of locoregional lymph nodes (negative nodes - N0) was found in 37 (37%) patients, while positive lymph nodes (N1 and N2) with present metastases were found in 63 (63%) of patients. The analysis of mean ADC values between the two mentioned subgroups did not reveal a statistically significant difference (p=0.357; p>0.05) (**Figure 5B**).

### 3.7 Mean ADC values in relation to TN stage

Based on data on local tumor extension and lymph node involvement (TN stage), patients were classified into the following stages: 19 patients with T2N0, 6 with T2N1, 1 with T2N2, 15 with T3N0, 17 with T3N1, 22 with T3N2, 3 with T4N0, 8 with T4N1 and 9 patients with T4N2. Analysis of the mean ADC values did not reveal a statistically significant difference depending on the different TN stage (p=0.196; p>0.05) (**Figure 5C**).

### 3.8 Mean ADC values in relation to local tumor status

Among patients with rectal cancer, the percentage of locally confined tumors (T1 and T2) was 26%, in contrast to locally advanced tumors (T3 and T4) which comprised 74%. Data processing did not reveal a statistically significant difference between ADC values in locally confined and locally advanced tumors (p=0.312; p>0.05) (**Figure 5D**).

### 3.9 Mean ADC values in relation to the presence of RAS mutation

Among patients with rectal cancer, molecular pathology for the presence of RAS mutation was performed in 19 patients, where the absence of RAS gene mutation was found in 9 patients, while 10 patients had a mutation of the mentioned gene. By comparing the mean ADC values between the mentioned subgroups, we did not find a statistically significant difference (p=0.829; p>0.05) (**Figure 5E**).

### 3.10 Mean ADC values in relation to the level of tumor markers

Twenty two patients had CEA tumor marker values above the reference range, while 22 patients had normal values of the mentioned oncomarker. An elevated concentration of the CA 19-9 tumor marker was found in 15 patients, while 25 patients had values of the mentioned tumor marker within the reference range. By analyzing the difference between patients with normal and elevated values of tumor markers, we did not find a statistically significant difference in the mean ADC values of rectal cancer (p=0.923; p>0.05) (**Figure 5F**).

## 4 Discussion

This study is the only one in the available literature that combined the entire spectrum of findings on the rectal wall, from normal findings to post-radiation proctitis to rectal cancer. The ADC values were observed in the mentioned conditions, all with the aim of more confident differentiation of rectal wall thickening in comparison to a normal wall.

Examining ADC values between the study groups, rectal cancer showed lower ADC values compared to both post-radiation proctitis and normal rectum. In comparison, post-radiation proctitis showed higher ADC values than normal rectum. At the same time, this is not the case with inflammation of the intestinal wall in inflammatory bowel diseases, where some authors show lower ADC values in relation to a normal intestinal wall (6). The explanation of this phenomenon lies in the fact that the free movement of water molecules is limited in hypercellular tumors (1), while in post-radiation proctitis there is damage to stem cells and atrophy of the mucosa with inflammation of the interstitium and edema (7), which facilitates the mentioned movement of water molecules and for this reason, the values in post-radiation proctitis are significantly different from the normal rectal wall, while lower ADC values in inflammation in inflammatory bowel diseases can be explained by the increased density of inflammatory cells in the wall itself, which leads to restriction of the diffusion of water molecules. For this reason, post-radiation proctitis is a special type of inflammation that is pathophysiologically different from other types of intestinal inflammation.

During the last few years, there has been increasing interest in using quantitative DWI parameters, such as ADC values, as biomarkers to predict the outcome of rectal cancer in relation to TN-stage and pathohistological characteristics, such as the degree of tumor differentiation and the presence of lymph node metastases.

In our research, there was no difference in the mean ADC values between different degrees of differentiation of rectal cancer (**Figure 5A**), which is in agreement with the results of other researchers (8–10), while on the other hand, Sun et al. (11) report significantly lower ADC values in high-grade cancers of the rectum in relation to low-grade ones. A possible explanation for the different results could be the almost three times lower number of patients with G1 and G3 grades in our study, in contrast to the study by Sun et al. (11). Also, a study by Liu et al. (12) highlights the importance of tumor texture analysis in order to determine the prognostic assessment of ADC values. Additionally, heterogeneity is an important characteristic of malignant lesions that originates from variations in tumor cellularity, angiogenesis, extravascular and extracellular matrix, as well as areas of hemorrhage and necrosis within the tumor, which further implies that greater tumor heterogeneity can lead to significant variations in ADC values (12).

By analyzing the mean ADC values in relation to the presence of infiltration of locoregional lymph nodes, we did not find a statistically significant difference indicative of metastasis (**Figure 5B**), which is in agreement with the results of previous studies (8, 12). In addition to the nodal status (N stage), we examined the local tumor status according to the T stage (**Figure 5D**). We found no significant difference in ADC values between patients with locally confined tumors (T1 and T2) versus locally advanced tumors (T3 and T4), which correlates with the results of the study by Liu et al. (12). We additionally analyzed the presence of a difference in ADC values depending on the different TN stage, but we did not obtain statistically significant results (**Figure 5C**). All of the above tells us that ADC is not a good marker for distinguishing the local tumor status, but on the other hand, it facilitates the diagnosis because it shows clear signs of diffusion restriction from the early stages (T1 and T2) which are clearly present in locally advanced stages (T3 and T4).

To date, few studies have investigated the association of DWI parameters in rectal cancer with different RAS proto-oncogene mutation status. One such study, by Xu et al. (13), states that the ADC values are significantly lower in the “KRAS-mutant” group compared to the group with the “KRAS wild-type” gene. Therefore, lower ADC values in the mutated group may indirectly confirm the association between KRAS mutation and prognosis in rectal cancer. On the other hand, a meta-analysis by Surov et al. (14) did not prove a statistically significant difference in ADC values in relation to KRAS gene mutation, which is confirmed by the results of our research (**Figure 5E**).

The level of tumor markers is potentially an important factor affecting the prognosis of the disease. However, after the analysis, the mean ADC values in patients with normal tumor markers did not differ significantly from the values in patients with elevated markers (**Figure 5F**). Our results are in agreement with the study of Sun et al. (11), which had a similar number of patients in each of the subgroups.

The research has several limitations. One of the potential limitations is that the examinations were performed on two different MR machines, magnetic field strengths 1.5T and 3T, although a study by Caruso et al. (15) did not show a statistically significant difference in ADC values between MR machines of different field strengths. However, this study mentioned that a 3T MRI provides superior detection of potential tumor residue compared to a 1.5T MRI, as the latter may produce less reliable ADC values (15). Another important limitation of the study is the absence of a definitive pathohistological finding in patients with locally advanced tumors in whom there was no possibility of surgical treatment during the course of the disease, and the local stage (primarily T and N stage) was determined based on MR examination. Additionally, the limitation of the study is the absence of ADC_msi_ and ADC_min_ values. These should be considered in future research, as some studies suggest that these values may be useful in assessing the aggressiveness of rectal cancer (16, 17). One of the most significant limitations of this study is the small sample size of certain subgroups, which may be why in several comparisons only a trend of increasing or decreasing values was observed, without statistically significant differences. Expanding the subgroups with smaller sample sizes in our study could reveal a statistically significant difference.

The advantage of this study is the unified presentation of the association of ADC values in relation to various pathohistological characteristics of rectal cancer and additional genetic and serological markers. Within this study, ADC values were examined in rectal adenocarcinoma, post-radiation proctitis and patients with normal rectum with a large sample, thus covering the spectrum of conditions that can be differential diagnostic problems. It is important to note that few studies looked at the ADC through the prism of RAS mutations and levels of tumor markers with a unique presentation of ADC values in patients with post-radiation proctitis and normal rectal wall. The ADC values can potentially help detect local recurrence following surgery or evaluate changes after chemoradiation therapy. These insights are crucial for clinicians, as they guide decisions on further diagnostic and therapeutic procedures, especially when evaluating suspicious thickening of the rectal wall, which is reflected in ADC values. Recent studies have identified various biomarkers that are useful in the diagnosis and prognosis of different carcinomas (18–21). Therefore, further research should aim to identify analogous radiological or other biomarkers and evaluate their impact on patient survival. Additionally, expanding the participant cohort and incorporating genetic parameters, as well as other biochemical markers associated with colorectal cancer, are crucial (22). Moreover, it is essential to investigate the potential of ADC as a prognostic biomarker and assess its impact on patient survival.

The ADC is a useful marker in differentiating between rectal cancer, post-radiation proctitis, and normal rectal wall with high sensitivity and specificity, but it cannot be used to distinguish the histological grades of rectal cancer, nor other pathohistological parameters like local tumor status, lymph nodes metastasis, TN stage and mutation of RAS gene, neither the level of tumor markers.

## Data Availability

The raw data supporting the conclusions of this article will be made available by the authors, without undue reservation.
